# Added Value of Diffusion-Weighted Magnetic Resonance Imaging in Differentiating Musculoskeletal Tumors Using Sensitivity and Specificity: A Retrospective Study and Review of Literature

**DOI:** 10.7759/cureus.12422

**Published:** 2021-01-01

**Authors:** Deb K Boruah, Bidyut Gogoi, Ruchi S Patni, Kalyan Sarma, Karuna Hazarika

**Affiliations:** 1 Radiodiagnosis, Tezpur Medical College, Tezpur, IND; 2 Radiodiagnosis, Assam Medical College, Dibrugarh, IND; 3 Pathology, Assam Medical College, Dibrugarh, IND; 4 Radiology, North Eastern Indira Gandhi Regional Institute of Health and Medical Sciences, Shillong, IND

**Keywords:** diffusion-weighted imaging (dwi), apparent diffusion coefficient (adc), bone tumor, soft tissue tumor

## Abstract

Background: Diffusion-weighted imaging (DWI) provides added value to conventional MRI imaging in diagnosing and differentiating various benign and malignant musculoskeletal tumors.

Objective: The study aims to evaluate the diagnostic efficacies of diffusion-weighted imaging along with the conventional MRI sequences for differentiating benign and malignant musculoskeletal tumors using sensitivity and specificity.

Materials and methods: This retrospective study was carried out on 73 histopathologically proven patients of various musculoskeletal tumors who presented to a tertiary care center between March 2017 to October 2018. Relevant clinical examinations and MRI scan of the requested body part of the musculoskeletal system were performed. Mean apparent diffusion coefficient (ADC) values were calculated in the bone as well as soft tissue tumors after placing uniform-sized region of interest (ROI) in the non-necrotic portion of the tumor.

Statistical analysis: Independent t-test and one-way analysis of variance (ANOVA) test were used to compare the mean ADC values of the various tumors with the histopathology. Receiver operating characteristic (ROC) curve analysis was done to determine the cut-off mean ADC values in the various bone and soft tissue tumors.

Results: Of 73 patients with musculoskeletal tumors (benign=20, malignant = 53), 47 patients were bone tumors (benign=12, malignant=35) and 26 patients were soft tissue tumors (benign=eight, malignant=18). Mean ADC value of benign bone tumor was 1.257±0.327[SD] x 10^-3^mm2/s and malignant was 0.951 ± 0.177[SD] x 10^-3^mm2/s. The mean ADC value of benign soft tissue tumor was 1.603±0.444[SD] x 10^-3^mm2/s and malignant was 1.036 ± 0.186[SD] x 10^-3^mm2/s. The cut-off mean ADC value was 1.058 x 10^-3^mm2/s for differentiating benign from malignant bone tumor with a sensitivity of 83.3%, specificity of 66.7% and accuracy of 78.7% while the cut-off mean ADC value of 1.198 x 10^-3^mm2/s for differentiating benign from malignant soft tissue tumors with a sensitivity of 83.3%, specificity of 87.5% and accuracy of 84.6%.

Conclusions: DWI with ADC mapping can be used as an additional reliable tool along with conventional MRI sequences in discriminating benign and malignant musculoskeletal tumors.

## Introduction

MRI is one of the imaging modalities of choice to detect intramedullary bony abnormality even with a negative bone scan [1]. Because of excellent soft-tissue contrast and multiplanar imaging capability, the conventional MRI sequences can delineate tumor size, margins, locations, tumor necrosis, and neurovascular bundle involvement, tumoral heterogeneity and adjacent joint involvement [2]. However, adding other MRI sequences like diffusion-weighted imaging (DWI), dynamic contrast-enhanced MRI (DCE-MRI), and MR spectroscopy helps to more accurately differentiate benign from malignant bone as well as soft tissue tumors [3-4].

DWI is a functional MRI technique based on the Brownian movement of water molecules where the apparent diffusion coefficient (ADC) quantifies the Brownian movement. The presence of cellular membranes limits the diffusion in a highly cellular microenvironment, resulting in low ADC value while free diffusion takes place in an acellular region resulting in high ADC value [5]. Because of this property, DWI is able to provide both qualitative and quantitative assessments of intra-tumoral cellularity [6]. Various literature have shown the added advantage of DWI and ADC mapping over the conventional MRI sequences in differentiating various musculoskeletal tumors, diffuse bone marrow infiltrative lesions, benign and pathological vertebral collapse [6-8]. DWI with ADC mapping is also utilized for assessment of the therapeutic response after treatment of various musculoskeletal or diffuse marrow infiltrative lesions [6-8]. Tumor response to radiotherapy or chemotherapy showed higher ADC values as compared with the pre-treatment ADC value [9]. DWI can be a reliable additional tool over conventional MR findings in differentiating different types of bone as well as soft tissue tumors [3,10-11]. In certain situations, conventional MRI fails to differentiate benign from malignant musculoskeletal tumors, where quantitative DWI, DCE-MRI, and MR spectroscopy play an important role [2-3,12-13]. Because of the overlapping of ADC values in the benign and malignant musculoskeletal tumors, tumor differentiation is complicated in a few situations as ADC values are affected by tumor cellularities and extracellular substances [14]. Previous studies concluded that DWI can be used as an adjunct imaging to conventional MRI sequences for characterization and differentiation of various musculoskeletal tumors [14-15].

The study aims to evaluate the diagnostic efficacies of diffusion-weighted imaging along with the conventional MRI sequences for differentiating benign and malignant musculoskeletal tumors using sensitivity and specificity.

## Materials and methods

Study design

A hospital-based retrospective study was conducted in a tertiary care hospital on 73 histopathologically confirmed patients with musculoskeletal tumors over for 18 months from March 2017 to October 2018. This retrospective study was approved by Institutional Ethics Review Committee.

Seventy-three histopathologically confirmed patients with various musculoskeletal tumors were included in this study after using the following exclusion criteria: 1. Benign soft tissue tumors like lipoma, angioleiomyoma, tenosynovial giant cell tumors were excluded because of false positivity due to their low ADC value. 2. Vertebral lesions. 3. Lesions with a diameter of less than 1 cm were excluded because of difficulties in placing a region of interest (ROI) for the measurement of ADC values. 4. Benign cartilaginous tumors like osteochondroma and enchondroma due to their high ADC value. 

Test methods

All patients underwent an MRI examination using a 1.5 T MR scanner (Magnatom Avanto; Siemens Medical Systems, Erlangen, Germany). An appropriate body or surface coil was used, depending upon the location and size of the lesion.

Various MRI sequences are shown in Table 1.

**Table 1:** showing parameters used in various MRI sequences for various musculoskeletal tumors DWI = Diffusion-weighted imaging (DWI), T1W = T1 weighted, T2W = T2 weighted, PDFS = proton density fat-suppressed

MRI sequence	TE(ms)	TR(ms)	Matrix	Slice thickness(mm)	Flip angle	Others
T1W sagittal / coronal	9-13	350-600	512 x 512	4	90^0^	
T2W axial, sagittal and coronal	75-90	3200-6300	512 x 512	4	90^0^	
Fat suppressed PDFS axial, sagittal and coronal	25-30	4500-4900	512 x 512	4	90^0^	TI=160ms
DWI axial or coronal	100-104	300-3500	128 x128	4	90^0^	b=0, 500, 1000sec/mm2
Fat-suppressed T1W post-contrast axial, coronal and sagittal	8-9	700-800	512 x 512	4	90^0^	after injecting I.V. Gadopentetate dimeglumine at a dose of 0.1mmol/kg body weight.

Analysis

Two radiologists retrospectively reviewed the MR images. During MR image analysis, the two radiologists were blinded to the clinical history or previous radiological reports of the studied patients. The size and ADC values were measured independently by the two radiologists and the mean values were used for the results.

Analysis of Conventional MR Images

We evaluated the following characteristics of a lesion like tumor sizes, margins, locations, neurovascular bundle involvement, peri-tumoral edema, tumor heterogeneity, and tumor necrosis. Tumor size was obtained from the largest dimension of a tumor. Margins of tumor classified into well defined, partially ill-defined, and ill-defined. A “well defined” margin was considered when the margin of a tumor was differentiated from surrounding structures regardless of peritumoral edema. A “partially ill-defined” was classified when the margin of a tumor was mostly well defined. The location of a tumor was classified as superficial when involving skin and subcutaneous tissue or deep when the tumor was located deeper in the deep fascia. The presence of bone involvement was confirmed when there was bony cortical erosion or medullary canal involvement. Peri-tumoral edema was defined where there were peri-tumoral bright T2 weighted images (T2WI) or proton density fat-suppressed (PDFS) hyperintensities. Tumor heterogeneity was defined with mixed-signal intensities on T1WI or T2W images. Tumor necrosis was defined as an area of T2WI or short tau inversion recovery (STIR) hyperintensities that were not enhanced on post-contrast images.

ADC Calculation Analysis

ADC values were generated on a pixel by pixel basis. Minimum, maximum and mean ADC values were calculated from placing either round or elliptical ROIs, however, mean ADC values were selected for statistical analyses. ADC values were expressed in 10-3 x mm2/second. We measured the ADC value in the operating system console using multiple uniform sizes (area, minimum 10 mm2, maximum 50 mm2) at least six ROIs, where three ROIs were placed in the central non-necrotic portion of the tumor and another three in the peripheral portion. Usually, the ROI was selectively placed in the solid, enhancing, non-necrotic, and/or DWI restricted regions of a tumor. The ROI position was always checked with reference to the conventional MRI images to avoid contamination from adjacent normal-appearing bone or soft tissue. The areas of artifacts, image distortions, partial volume effect, and most peripheral margin of a tumor were avoided for ROI placement. In patients with multiple bony or soft tissue tumors, the largest lesion was selected for calculation of the mean ADC value.

Histopathology

Histopathological diagnosis of musculoskeletal tumors was established on surgically resected specimens in 29 patients, core needle biopsy specimens in 41 patients, and fine-needle aspiration (FNAC) specimens in three patients. Ultrasound-guided (USG) or CT-guided core needle biopsy specimens were obtained by using a 16-18 gauge core biopsy needle (BARD Biopsy System; Tempe, Arizona, USA). MRI studies were always performed before the biopsy or aspiration procedure. Biopsy or aspiration procedures were done from the enhancing, non-necrotic, and diffusion-restricted portion of a tumor.

Statistical Analysis

All statistical analysis was performed using Statistical Package for Social Sciences (SPSS) version 16 (IBM Corp., Armonk, NY, USA). The clinical data and different parameters of conventional MR imaging in bone and soft tissue tumors were evaluated with a chi-square test. The strength of association between the mean ADC values with the nature of tumor on histopathology was assessed using an independent t-test and one-way analysis of variance (ANOVA) test. Optimal cut-off mean ADC values of various musculoskeletal tumors were obtained from receiver operating characteristic (ROC) curve analysis.

## Results

**Figure 1:**
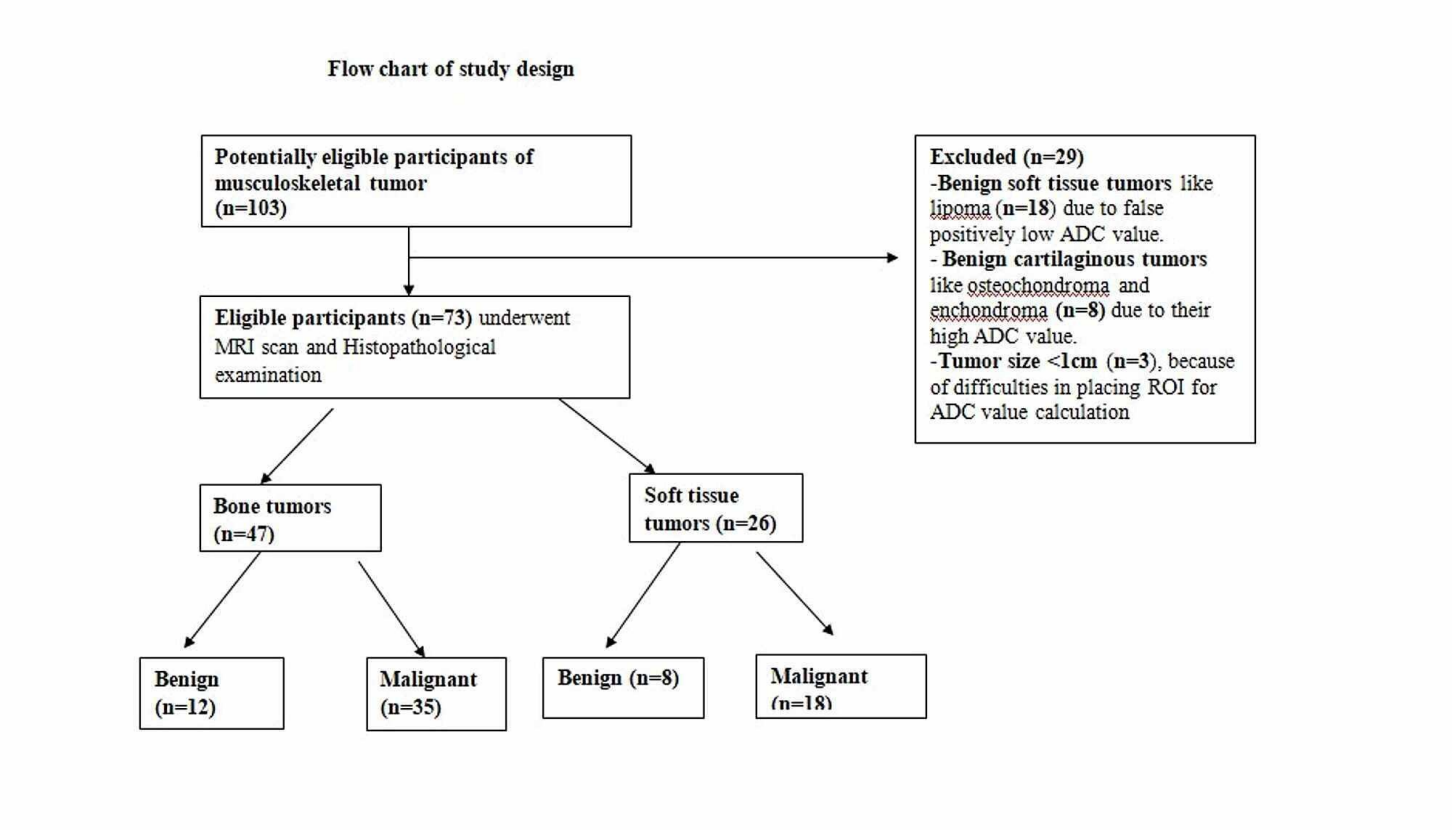
Flow chart of study design

Seventy-three histologically proven patients of musculoskeletal tumors were included in this hospital-based retrospective study (Figure 1).

The study sample comprised 73 patients of musculoskeletal tumors (bone=47, soft tissue=26). The various histopathological types of bone and soft tissue tumors are shown in Table 2.

**Table 2:** Types of bone and soft tissue tumors in 73 patients

Benign tumors (n=20)	Number (n=20)	Malignant tumors (n=53)	Number(n=53)
Giant cell tumor of bone	11	Bony metastasis	14
Schwannoma	5	Soft tissue sarcoma	10
Fibromatosis	2	Osteosarcoma	5
Chondromyxoid fibroma	1	Fibrosarcoma	5
Fibroma	1	Malignant fibrous histiocytoma	5
		Ewing sarcoma	4
		Liposarcoma	3
		Lymphoma1	2
		Chondrosarcoma	2
		Ameloblastoma	1
		Plasmocytoma	1
		Malignant Haemangiopericytoma	1

The anatomical locations of the tumors are shown in Table 3.

**Table 3:** The anatomical location of various bone and soft tissue tumors in 73 patients.

Nature of tumor	Anatomical location	Benign	Malignant
Bone tumor	Lower extremity	9	20
	Upper extremity	1	7
	Buttock and pelvic area	1	7
	Back and chest wall	-	1
Soft tissue tumor	Lower extremity	2	8
	Upper extremity	1	9
	Buttock and pelvic area	2	0
	Back and chest wall	3	1

Results of clinical data and conventional MR images

Patient age, tumor margin, tumor necrosis, and adjacent joint involvement were found to be significantly related to the ability to differentiate benign and malignant bone tumors, but gender, neurovascular bundle involvement, peri-tumoral edema, and tumor heterogeneity were not (Table 4).

**Table 4:** showed the results of clinical data and Conventional MR imaging findings in bone tumors

Parameters		Benign tumors (n=12)	Malignant tumors (n=35)	p-value
Age (years)		29.4±8.8[SD]	43.8±2.1[SD]	0.001
Sex		M:F=7:5	M:F=25:10	0.473
Tumor size(cm)		8.3±5.6[SD]	11±5.8[SD]	0.001
Tumor margin				0.001
	Well- defined	6(50%)	3(8.6%)	
	Partially ill-defined	5(41.7%)	20(57.1%)	
	Ill -defined	1(8.3%)	12(34.3%)	
Neurovascular bundle (NVB)				0.146
	No involvement	4(33.3%)	10(28.6%)	
	Displacement	7(58.3%)	16(45.7%)	
	Encasement	1(8.3%)	9(25.7%)	
Peri-tumoral edema				0.096
	Yes	7(58.3%)	33(94.3%)	
	No	5(41.7%)	2(5.7%)	
Tumor Heterogeneity				0.569
	Yes	7(58.3%)	30(85.7%)	
	No	5(41.7%)	5(14.3%)	
Tumor Necrosis				0.044
	Yes	4(33.3%)	23(65.7%)	
	No	8(66.7%)	12(34.3%)	
Adjacent joint involvement				0.007
	No involvement	5(41.7%)	21(60%)	
	Involvement	7(58.3%)	13(37.1%)	

Patient age, tumor size, margin, peri-tumoral edema, neurovascular bundle involvement, tumor heterogeneity, and tumor necrosis showed statistical significance to differentiate benign from malignant soft tissue tumors (Table 5).

**Table 5:** showed the results of clinical data and Conventional MR imaging findings in soft tissue tumors

Parameters		Benign tumors (n=8)	Malignant tumors (n=18)	p-value
Age (years)		25.7±14.8[SD]	37.8±16.8[SD]	0.001
Sex		M:F=3:5	M:F=9:9	0.083
Tumor size(cm)		9.4 ±4[SD]	10±5.9[SD]	0.001
Tumor margin				0.001
	Well- defined	5(62.5%)	5(27.8%)	
	Partially ill-defined	3(37.5%)	8(44.4%)	
	Ill -defined		5(27.8%)	
Location				0.001
	Superficial	1(12.5%)	3(16.7%)	
	Deep	5(62.5%)	8(44.4%)	
	Both	2(25%)	7(38.9%)	
Neurovascular bundle (NVB)				0.029
	No involvement	3(37.5%)	3(16.7%)	
	Displacement	5(62.5%)	9(50%)	
	Encasement		6(33.3%)	
Peri-tumoral edema				0.032
	Yes	2(25%)	9(50%)	
	No	6(75%)	9(50%)	
Tumor Heterogeneity				0.032
	Yes	2(25%)	9(50%)	
	No	6(75%)	9(50%)	
Tumor Necrosis				0.001
	Yes		6(33.3%)	
	No	8(100%)	12(66.7%)	
Bone involvement				0.003
	No involvement	6(75%)	13(72.2%)	
	Involvement	2(25%)	5(27.8%)	

**Figure 2:**
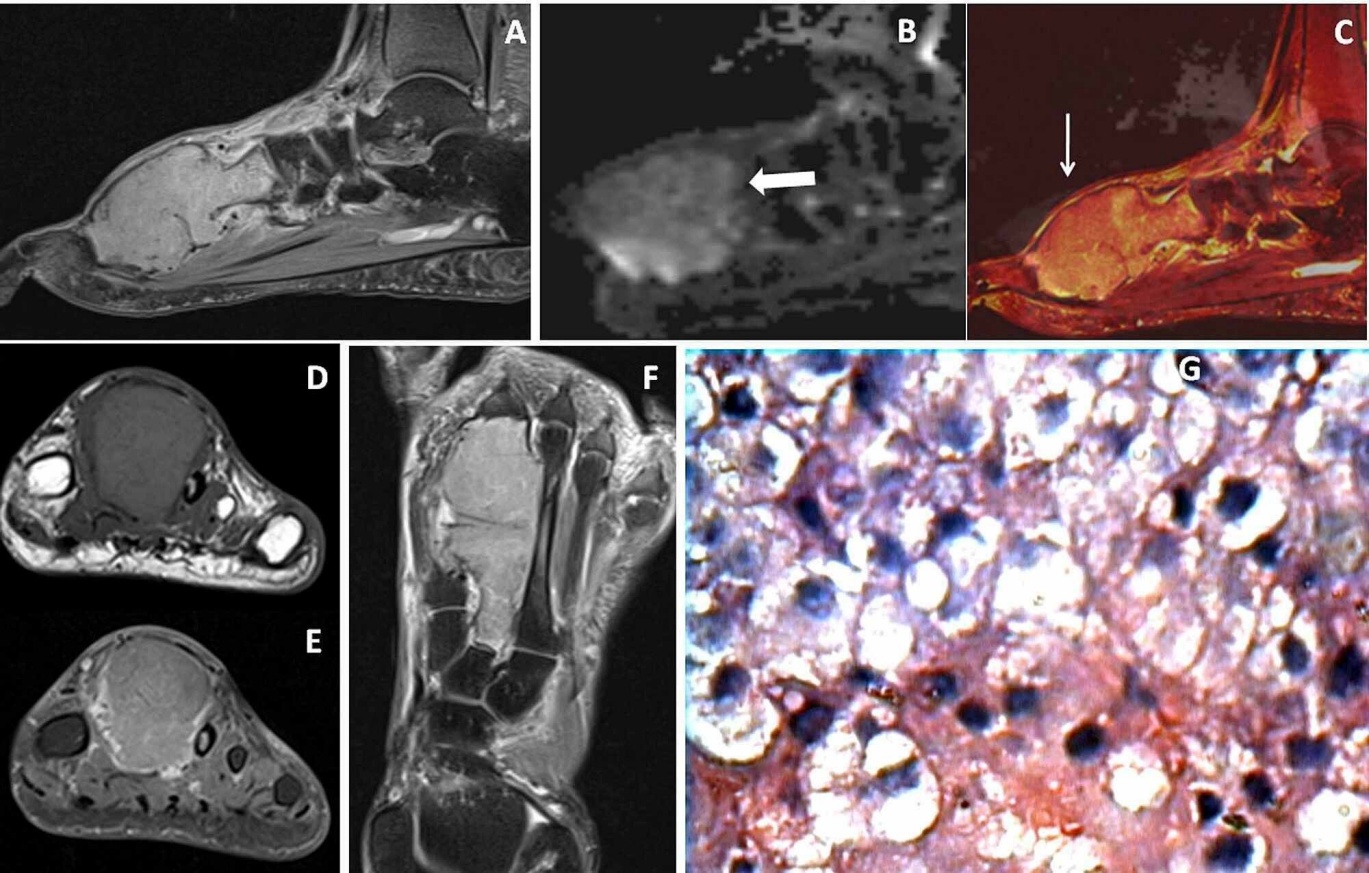
Acral metastasis Sixty-six-year-old male had swelling in his left foot. Sagittal proton density fat-suppressed (PDFS) image (A) showed a hyperintense expansile mass lesion in the second metatarsal bone extending into articular surfaces. Sagittal diffusion-weighted image (DWI) (B) showed diffusion restriction (←block arrow) with bright signals on fusion image (C) between sagittal PDFS and DWI (↓ arrow). Coronal T1 weighted (T1W) image (D) showed expansion of the affected secondary metatarsal with pressure erosion over the third metatarsal bone. Coronal and axial post gadolinium fat-suppressed T1W images (E&F) showed moderate homogenous enhancement of the mass. 40X HPE (image G) showed metastasis.

**Figure 3:**
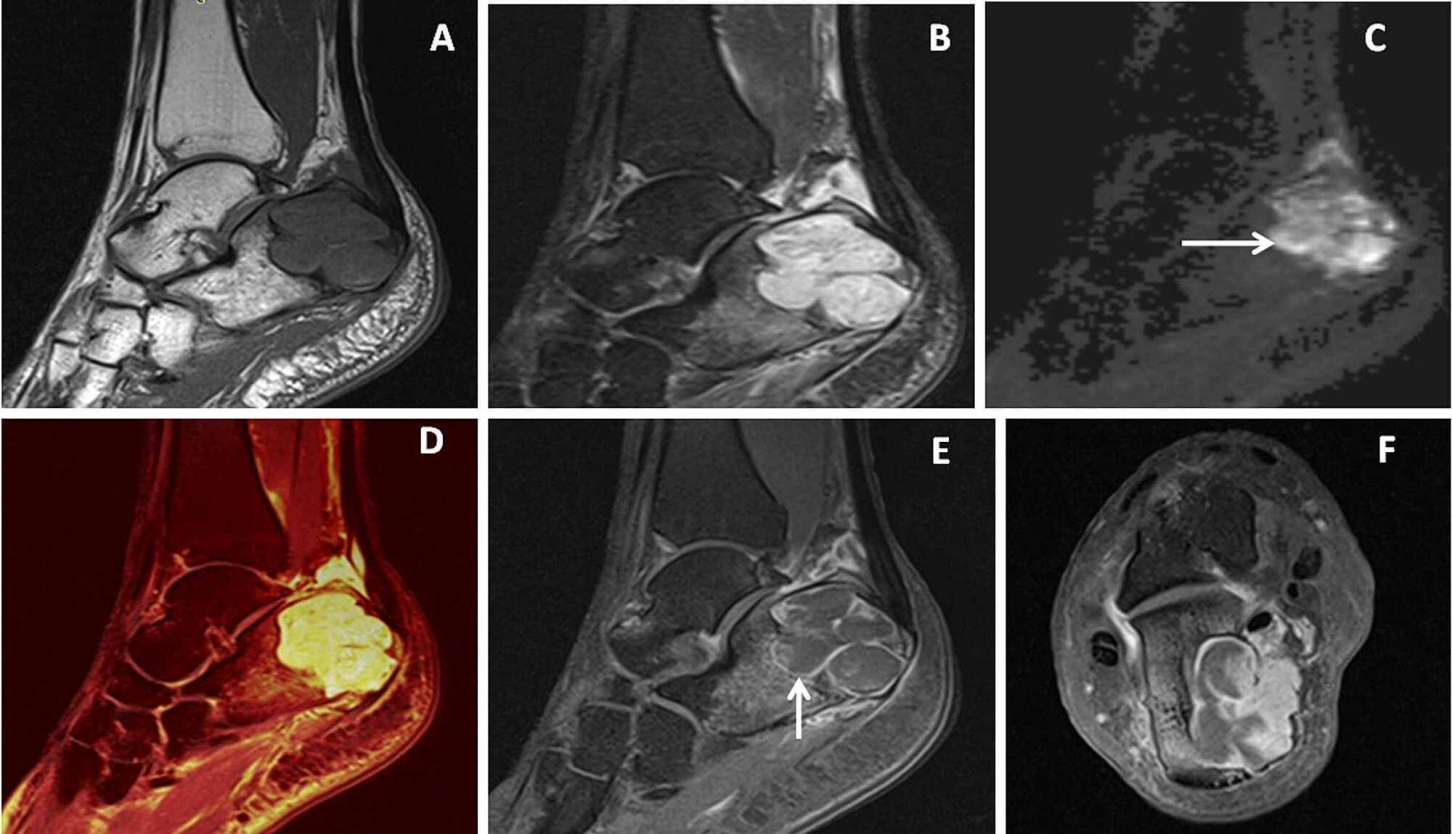
Calcaneal giant cell tumor (GCT) Thirty-six-year-old female with calcaneal GCT. Sagittal T1 weighted image (T1WI) and proton density fat-suppressed (PDFS) images (A &B) showed T1WI hypo and PDFS hyperintense expansile lesion in the posterior portion of calcaneum. Sagittal diffusion-weighted imaging (DWI) (C) showed diffusion restriction (→ arrow), where fusion image (D) between sagittal PDFS and DWI showed bright signals within the lesion. Post gadolinium fat-suppressed sagittal and axial T1W images (E &F) showed moderate heterogeneous enhancement of the mass with more irregular peripherally enhancing solid components with enhancing septae (↑ arrow ).

**Figure 4:**
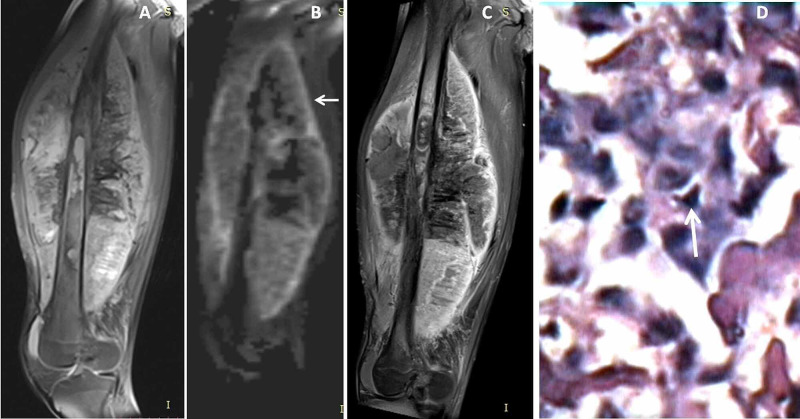
Diaphyseal osteosarcoma Eighteen-year-old male with right thigh swelling. Sagittal proton density fat-suppressed (PDFS) image (A) showed a large lobulated mixed signal intensity mass lesion around the femoral diaphysis with intramedullary altered signal intensities and hypointense sunray appearing new bone formations. Sagittal diffusion-weighted imaging (DWI) (B) showed peripheral dominant diffusion restriction (←arrow). Fat-suppressed post gadolinium sagittal T1 weighted (T1W) image (C) showed heterogeneous enhancement of the mass with areas of necrosis and sunray appearing new bone formation had adjacent soft tissue infiltrations. 40X HPE image (D) showed pleomorphic cells (↑ arrow) with osteoid formations suggesting osteosarcoma.

**Figure 5:**
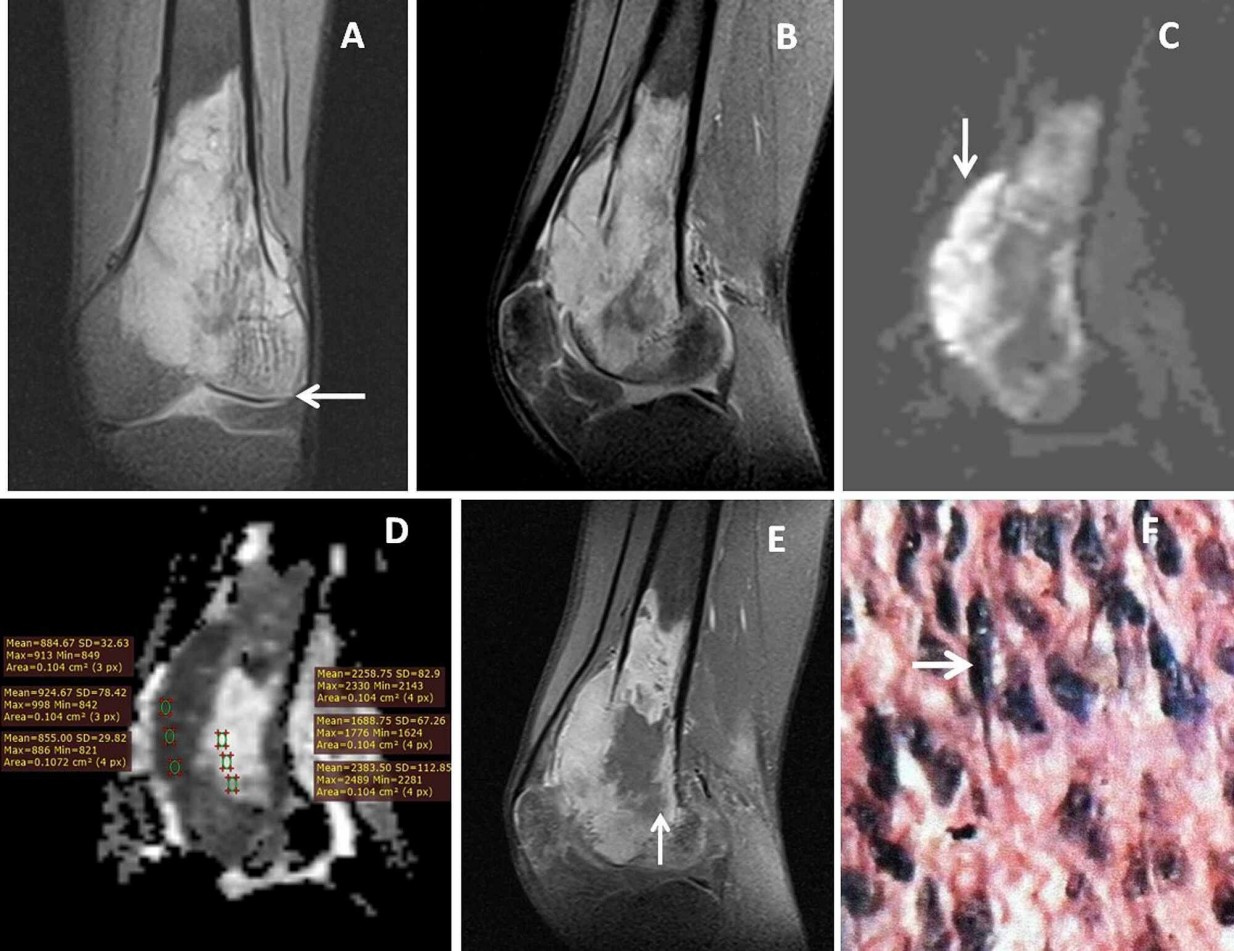
Distal femoral fibrosarcoma Twenty-year-old male had swelling around the left knee joint. Coronal and sagittal fat-suppressed proton density (PDFS) image (A & B) showed eccentric hyperintensities in distal meta-diaphyseal region of femur with epiphyseal extension into lateral femoral condyle ( ← arrow) with destruction of cortices. Sagittal diffusion-weighted imaging (DWI) image (C) with b value 1000s/mm2 showed irregular patchy peripheral diffusion restrictions (↓arrow) where apparent diffusion coefficient (ADC) image (D) showed low ADC value in peripheral and high in central portion of the tumor. Post-gadolinium image (E) showed heterogeneous enhancement of the mass with central necrosis (↑arrow). 40X HPE image (F) showed highly pleomorphic cells ( →arrow) with features of fibrosarcoma.

**Figure 6:**
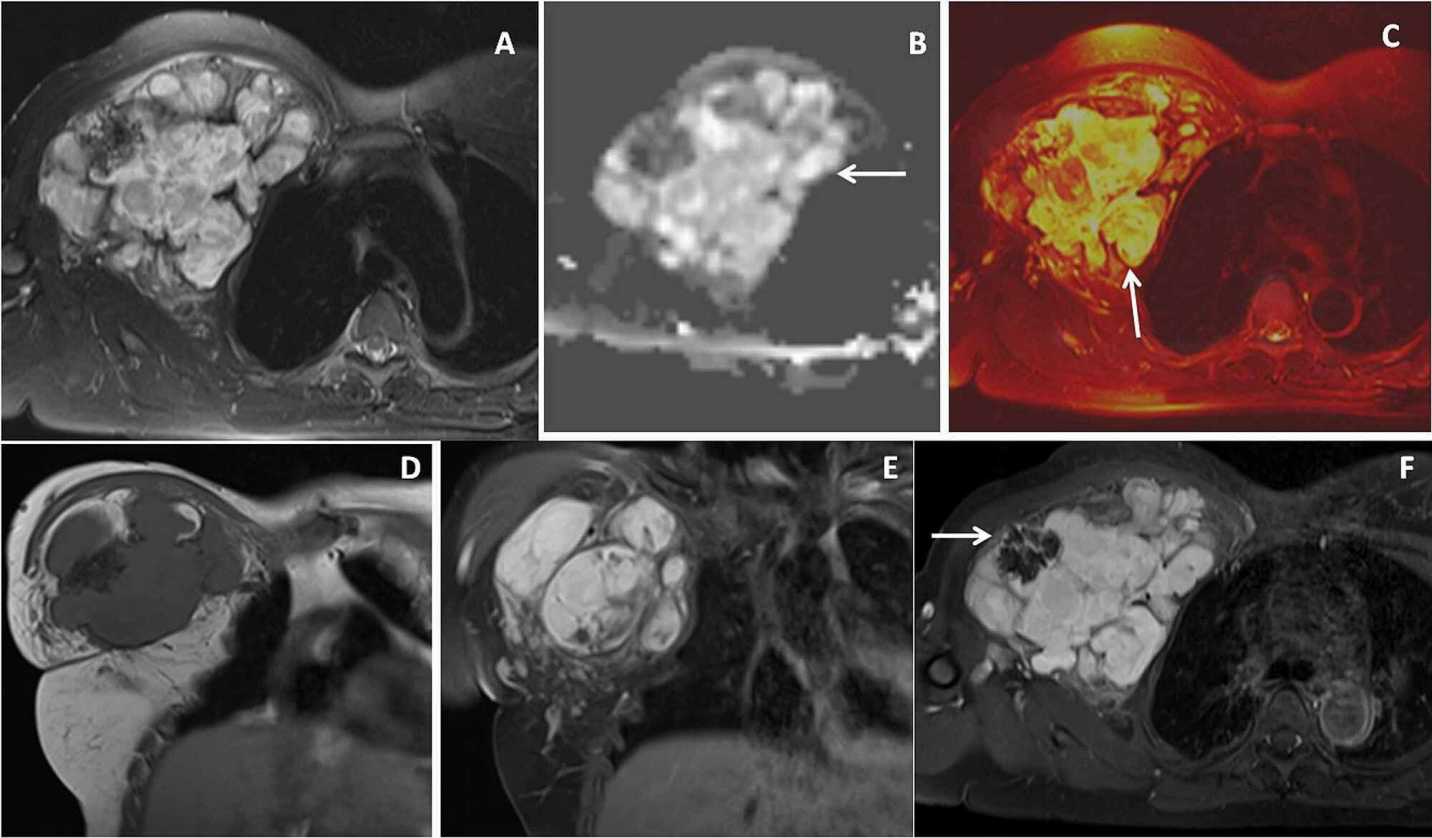
Chest wall malignant fibrous histiocytoma Thirty-six-year-old female with malignant fibrous histiocytoma (MFH) of right chest wall. Axial proton density fat-suppressed (PDFS) image (A) showed a larger lobulated heterogeneously hyperintense mass lesion in the right upper anterior chest wall causes anterior displacement of the pectoralis muscle. Axial diffusion-weighted image (DWI) (B) image shows bright signals within the mass (← arrow) while fusion image (C) between axial PDFS and DWI showed the bright signals in major portions of lesion (↑arrow). Coronal T1 weighted image (T1WI) (D) shows T1WI isointense signal intensities of lesion with ill-defined T1W hypointensities within. Fat-suppressed post gadolinium coronal and axial T1W images (E & F) showed heterogeneous enhancement of the lesion with non-enhancing T1 low signal intensity lesion(→arrow).

**Figure 7:**
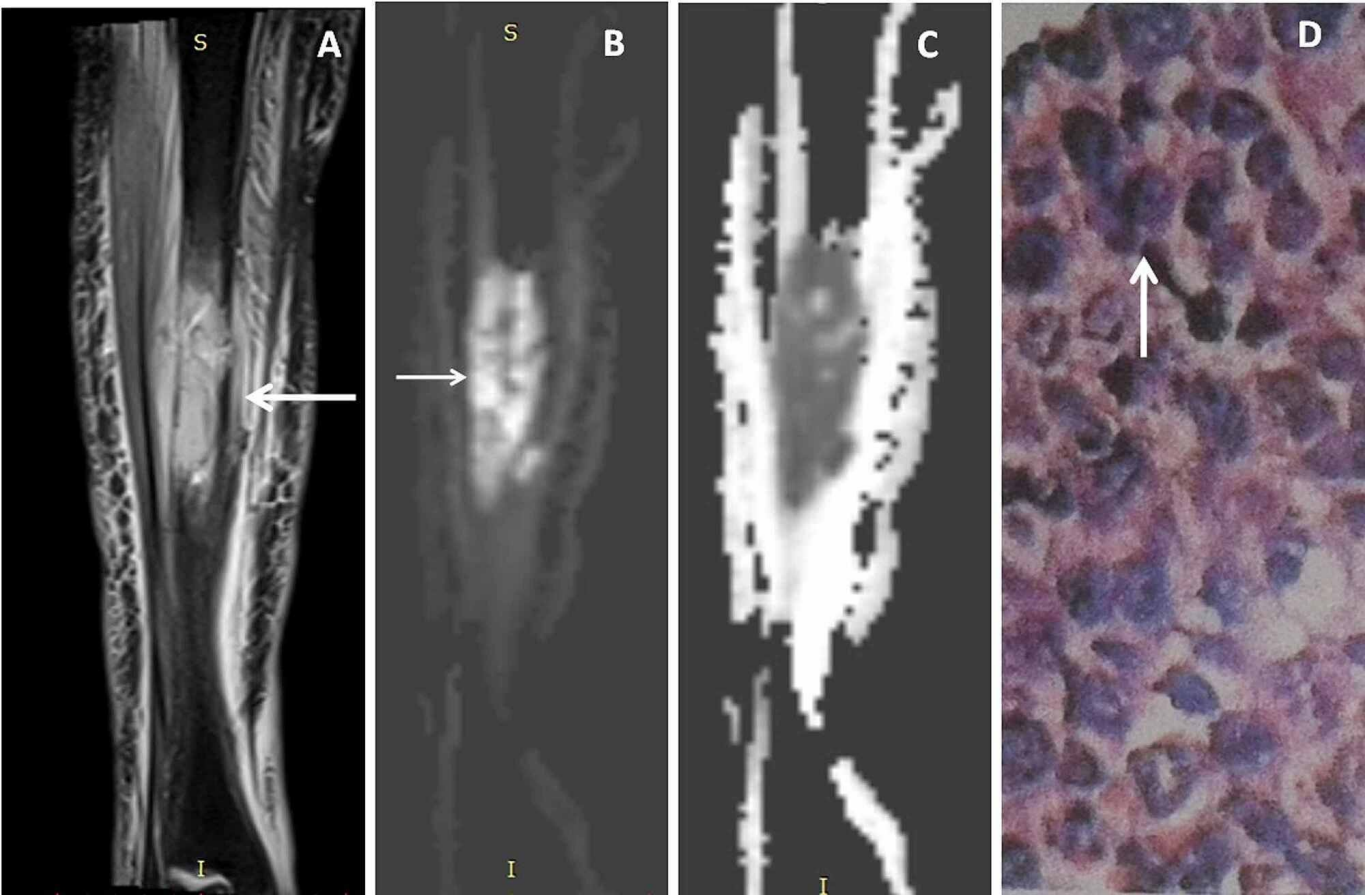
Tibial shaft lymphoma Forty-seven-year-old female with right leg swelling. Coronal proton density fat-suppressed (PDFS) image (A) showed minimally expansile intramedullary lesion in middle 3rd of tibial shaft with destructions of bony cortices and surrounding soft tissue infiltrations (←arrow). Coronal diffusion-weighted image (DWI) (B) and apparent diffusion coefficient (ADC) map image (C) showed diffusion restriction (→ arrow) with low ADC value. 40X HPE image (D) showed bony tissue infiltrated by tumor composed of small round to oval cells, (↑ arrow) where the cells showed vesicular nuclei with prominent nucleoli. Immunehistochemistry confirmed diagnosis of non-Hodgkin's lymphoma with positive leucocyte common antigen (LCA) and negative cytokeratin and desmine.

**Figure 8:**
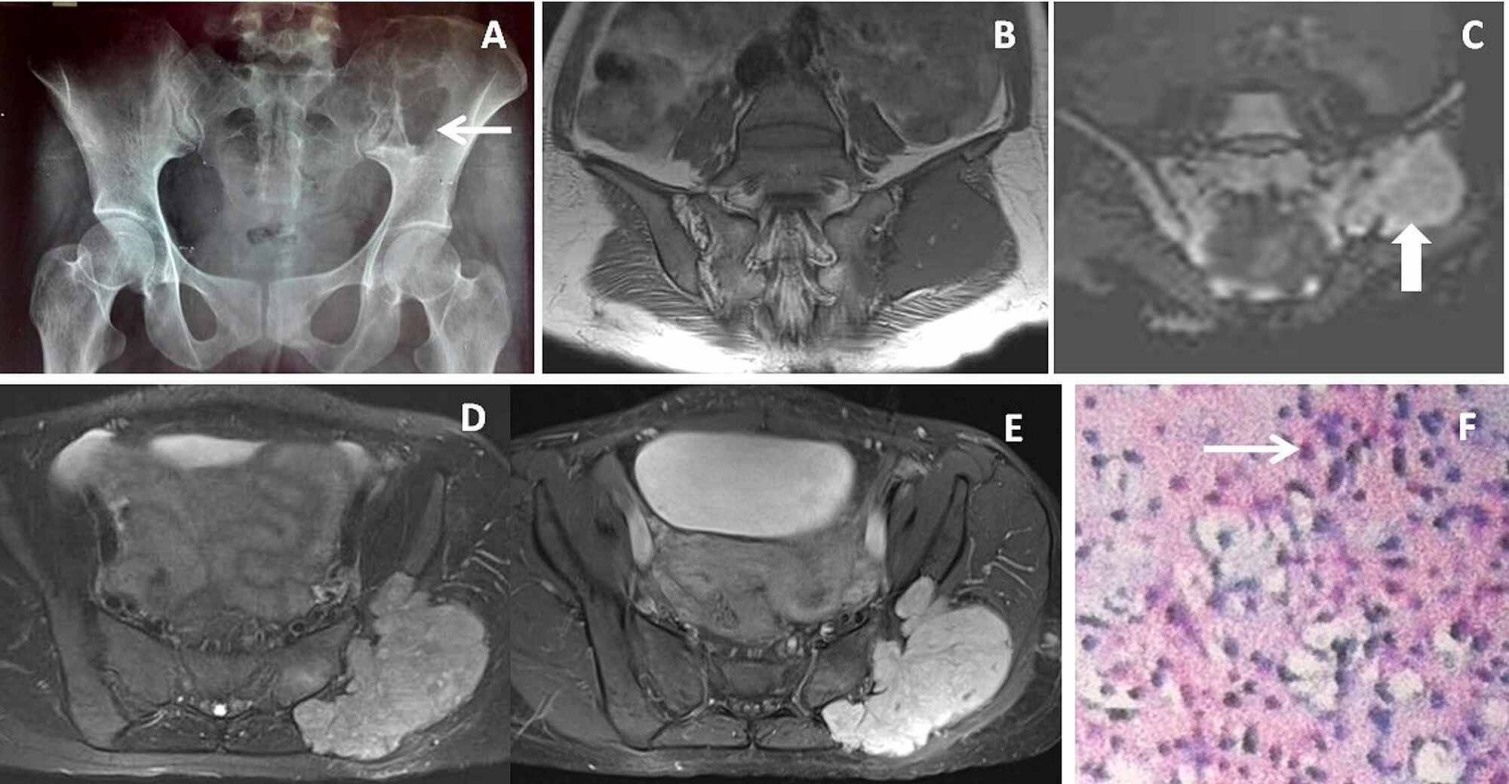
Iliac bone chondromyxoid fibroma Thirty-two-year-old female presented with pain and swelling in left buttock for two years. X-ray AP view of pelvis (A) showed ill-defined osteolytic lesion in left iliac bone (← arrow). Coronal T1 weighted (T1W) image  B ) showed a well-defined lobulated hypointense lesion in the left iliac blade. Coronal diffusion-weighted image (DWI) (C) shows diffusion restriction within the mass (↑ arrow). Axial proton density fat-suppressed (PDFS) image (D) showed the lobulated hyperintense lesion in the left iliac blade displacing adjacent gluteal muscles. Post gadolinium axial T1W image (E ) showed moderate homogenous enhancement of the lesion. 10X HPE image (F) showed lobules of spindle-shaped cells in abundant myxoid to chondroid stroma ( →arrow) suggesting chondromyxoid fibroma.

Table 4 and Table 5 summarize the clinical data and conventional MRI findings in various bone and soft tissue tumors. Bone tumors were observed in 47 patients (benign=12, malignant=35) and soft tissue tumors in 26 patients (benign=eight, malignant=18). Out of 73 patients, 14 patients had metastatic tumors (Figure 2), 11 patients giant cell tumors (GCT) (Figure 3), 10 patients soft tissue sarcoma, five patients each had osteosarcoma (Figure 4), fibrosarcoma (Figure 5), malignant fibrous histiocytoma (Figure 6) and schwannoma, four patients had Ewings sarcoma, three patients had liposarcoma, two patients each had lymphoma (Figure 7), chondrosarcoma and fibromatosis, and one patient each had haemangioendothelioma, chondromyxoid fibroma (Figure 8), plasmocytoma, malignant haemangiopericytoma and fibroma.

Results of quantitative diffusion-weighted imaging

**Figure 9:**
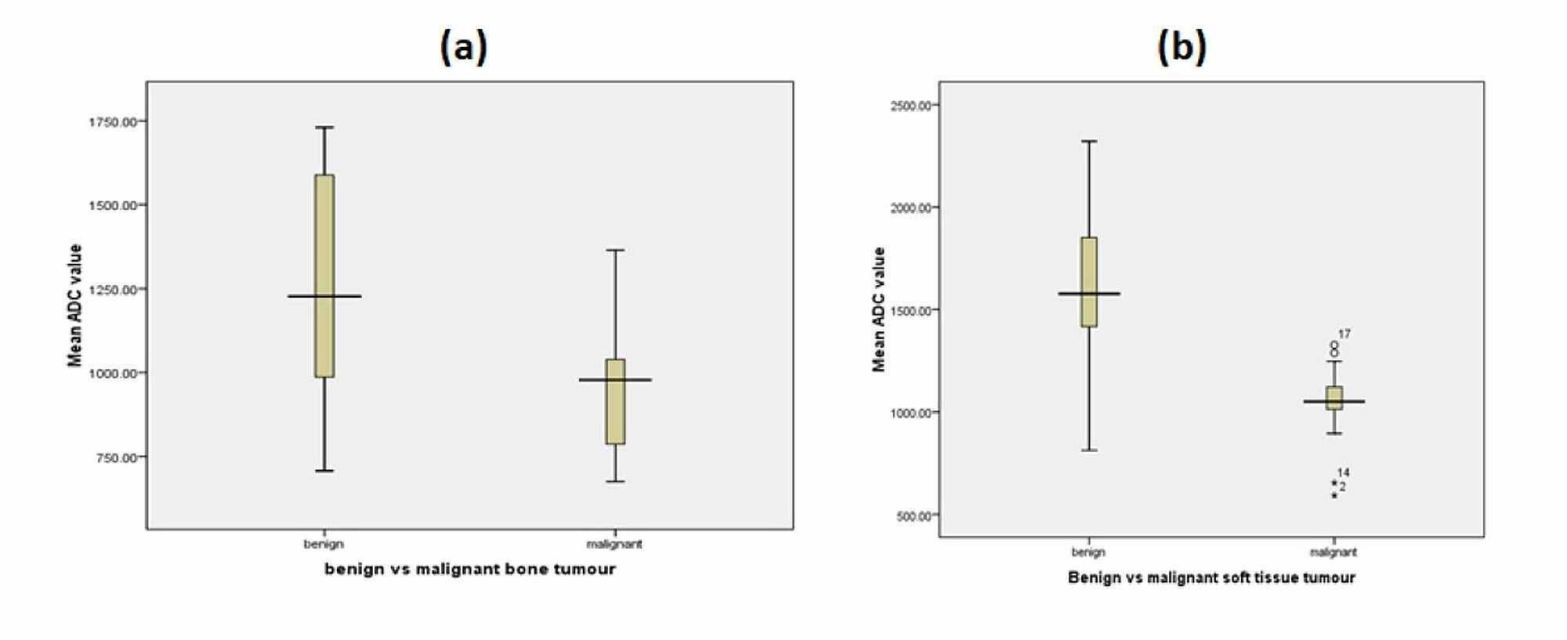
Boxplot summarizing the range of distribution of the mean apparent diffusion coefficient (ADC) values of bone and soft tissue tumors in 73 patients. (a) shows a boxplot of mean ADC value of benign and malignant bone tumors show a considerable overlap between these two groups. Despite this overlap, there was a statistically significant difference between the mean ADC value of benign and malignant bone tumors with a p-value of 0.001. (b) shows a boxplot of mean ADC value of benign and malignant soft tissue tumors with a statistically significant difference between the mean ADC value with a p-value of 0.001.

The mean ADC value of bone tumor was 0.996 ± 0.269[SD] x 10-3mm2/s and soft tissue tumor was 1.216 ± 0.390[SD] x 10-3mm2/s. There was a statistically significant difference between the mean ADC value of bone and soft tissue tumors (p-value 0.006) using the unpaired student t-test. The mean ADC value of benign bone tumor was 1.257±0.327[SD] x 10-3mm2/s and malignant bone tumor was 0.951 ± 0.177[SD] x 10-3mm2/s with a statistically significant difference between the mean ADC value of benign and malignant bone tumors (p-value 0.001). The mean ADC value of benign soft tissue tumor was 1.603±0.444[SD] x 10-3mm2/s and malignant soft tissue tumor was 1.036 ± 0.186[SD] x 10-3mm2/s with a statistically significant difference between the mean ADC value of benign and malignant soft tissue tumors (p-value 0.01) using the unpaired student t-test. The distribution of the mean ADC value of bone and soft tissue tumors is shown in the boxplot (Figure 9).

Table 6 shows the mean ADC value of musculoskeletal tumors in our study sample.

**Table 6:** summarized the mean apparent diffusion coefficient (ADC) value of commonly encountered musculoskeletal tumors in 73 patients.

Tumor	Number (%)	Mean ADC (x 10-3 mm2/s)
Bone Tumors	47/73(64.4%)	0.996 ± 0.269[SD]
Soft tissue tumor	26/73(35.6%)	1.216 ± 0.390[SD]
Benign bone tumor	12/47(25.5%)	1.257±0.327[SD]
Malignant bone tumor	35/47(74.5%)	0.951 ± 0.177[SD]
Benign soft tissue tumor	8/26(30.8%)	1.603±0.444[SD]
Malignant soft tissue tumor	18/26(69.2%)	1.036 ± 0.186[SD]
Bony Metastasis	14/73(19.2%)	0.939±0.109[SD]
Soft tissue sarcoma	10/73(13.7%)	1.081±0.234[SD]
Giant cell tumor (GCT)	11/73(15.1%)	1.251±0.342[SD]
Osteosarcoma	5/73(6.8%)	0.782±0.125[SD]
Malignant Fibrous Histiocytoma	5/73(6.8%)	0.916±0.159[SD]
Fibrosarcoma	5/73(6.8%)	0.938±0.162[SD]
Ewing sarcoma	4/73(5.5%)	0.689±0.087 [SD]
Liposarcoma	3/73(4.1%)	1.125±0.029 [SD]
Schwannoma	5/73(6.8%)	1.440±0.363[SD]
Chondrosarcoma	2/73(2.7%)	1.324±0.048[SD]
Lymphoma	2/73(2.7%)	0.747±0.315[SD]

ROC curve analysis of ADC mapping

**Figure 10:**
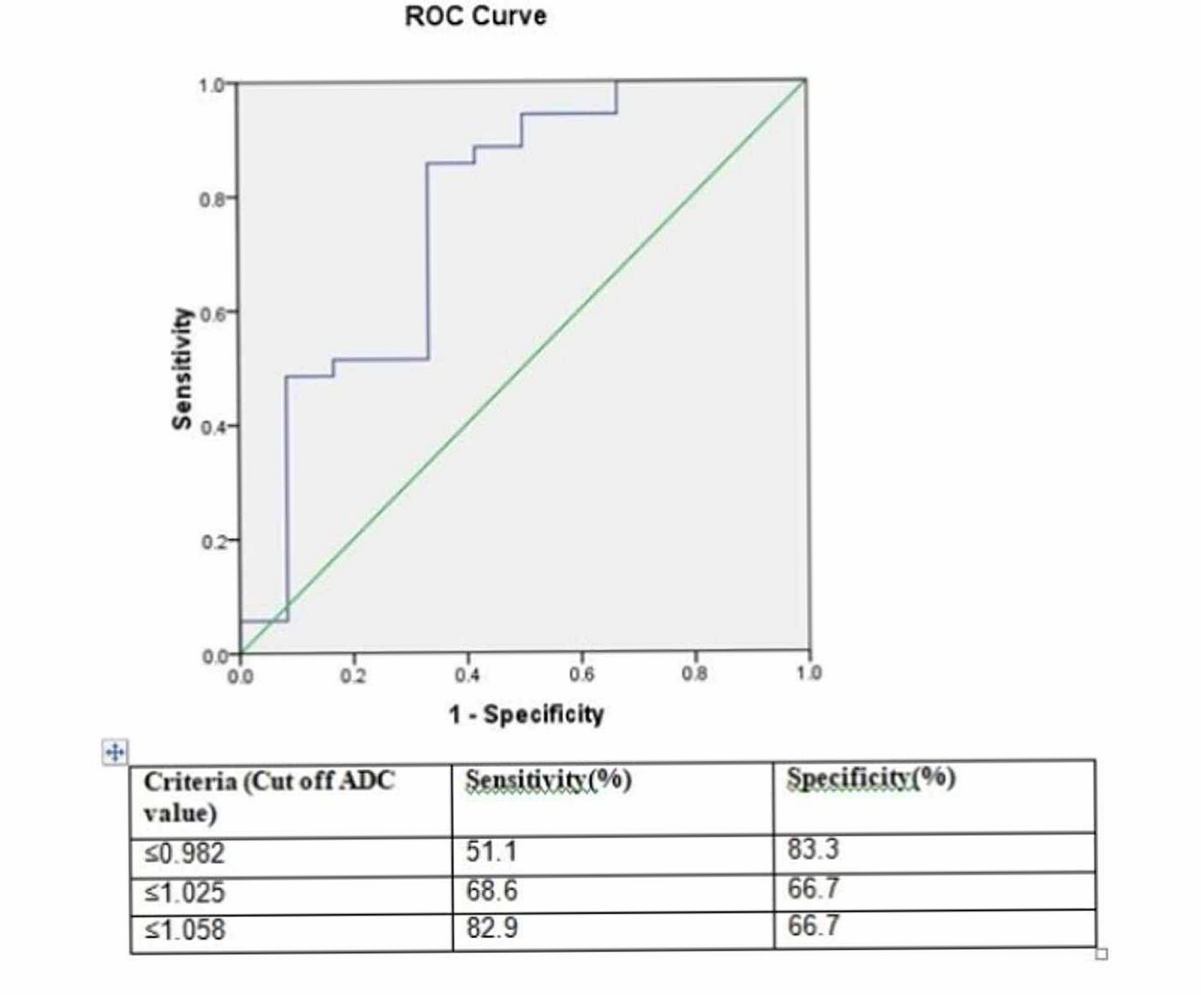
Receiver operating characteristic (ROC) curve of mean apparent diffusion coefficient (ADC) value of bone tumors in 47 patients.

Receiver operating characteristic (ROC) curve analysis showed cut-off mean ADC value of 1.058 x 10-3mm2/s for differentiating benign from malignant bone tumor with a sensitivity of 83.3%, specificity of 66.7%, positive predictive value of 87.8%, negative predictive value of 57.1% and a diagnostic accuracy of 78.7% (Figure 10).

**Figure 11:**
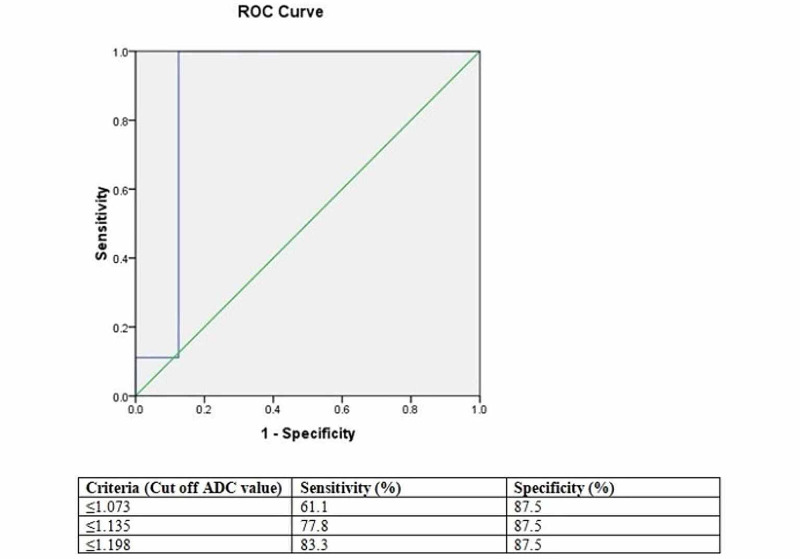
Receiver operating characteristic (ROC) curve of mean apparent diffusion coefficient (ADC) value of soft tissue tumors in 26 patients.

However four patients (8.5%) of giant cell tumor (GCT) showed ADC value lower than the cut-off value of 1.058 x 10-3mm2/s while another one patient (2.1%) of chondrosarcoma showed ADC value higher than the cut-off ADC value. Receiver operating characteristic (ROC) curve analysis showed cut off mean ADC value of 1.198 x 10-3mm2/s for differentiating benign from malignant soft tissue tumor with a sensitivity of 83.3%, specificity of 87.5%, positive predictive value of 93.7%, negative predictive value of 70% and a diagnostic accuracy of 84.6% (Figure 11).

However two patients (7.7%) of soft tissue sarcoma showed ADC value higher than the cut-off ADC value of 1.198 x 10^-3^mm2/s while another one patient (3.8%) of schwannoma showed ADC value lower than cut-off ADC value.

## Discussion

Quantitative diffusion-weighted imaging (DWI) and ADC mapping had a valuable role in characterizing and differentiating various bone and soft tissue tumors, which may improve the diagnostic accuracy in addition to the conventional MR imaging [14]. The non-contrast MRI techniques like DWI and MR Spectroscopy can be used in situations where intravenous contrast media is contraindicated. Usually, malignant tumors have low ADC values and benign tumors have high ADC values with some exceptions like giant cell tumor (GCT) and osteoblastoma, which show low ADC values [14]. The low ADC values in GCTs are due to the reduction of extracellular space from histiocytes, multi-nucleated giant cells, hemosiderin granules, and collagenous strands [3,16-17]. High ADC values were observed in chondroid lesions, probably due to high free extracellular water content, regardless of their cellularity and histological grading [18-20]. The malignant chondroid tumors usually show higher ADC values than benign chondroid tumors [17] because of the high content of the chondroid matrix [16-17]. Ewing sarcoma shows the lowest ADC value in the group of sarcomas [21].

Quantitative ADC mapping helps in differentiating residual or recurrent tumor in post-treated (post-irradiated) or post-operated musculoskeletal tumors [22]. Post-treated tumors with areas of higher ADC value than the pretreatment value indicate tumor cell necrosis which suggests a positive response to the therapy [6,22]. Some previous studies showed that post-treatment increasing ADC value in primary bone sarcoma correlated well with successful treatment [7,23].

In our study, conventional MR imaging parameters like tumor margin, tumor necrosis, and adjacent joint involvement were found to be significantly related to the ability to differentiate benign and malignant bone tumors, but neurovascular bundle involvement, peritumoral edema, and tumor heterogeneity were not. But most of the conventional MRI parameters like tumor size, margin, peri-tumoral edema, neurovascular bundle involvement, tumor heterogeneity, and tumor necrosis showed statistical significance to differentiate benign from malignant soft tissue tumor. So quantitative DWI imaging helps as an additional reliable tool in the evaluation of musculoskeletal tumors, especially bone tumors. When low ADC values and malignant MRI characteristics are combined, more accurate results are obtained in our study sample as compared to evaluating only ADC values.

In our study sample, considerable ADC value overlap was noted while differentiating various benign and malignant bone as well as soft tissue tumors. Even though in our study sample, 29 (82.8%) out of 35 patients of malignant bone tumor showed lower ADC value below the cut-off value of 1.058 x 10-3mm2/s while 15 (83.3) out of 18 patients of malignant soft tissue tumor showed lower ADC value below the cut-off value of 1.198 x 10-3mm2/s. But we do not know whether this ADC value overlapping is mainly due to heterogeneity of various bone and soft tissue tumor subtypes or other factors. However, our study findings of cut-off ADC values add further support to previously published studies [6,24]. Our study sample showed a cut-off mean ADC value of 1.198 x 10-3mm2/s for differentiating benign and malignant soft tissue tumor with a sensitivity of 83.3%, specificity of 87.5% and these findings are well correlated with the previous study of Choi et al. [25] who found cut-off mean ADC value of 1.18 x10-3 mm2/sec with a sensitivity of 86.11% and specificity of 77.05%, Hassanien et al. who found cut-off ADC value of 1.235 x 10-3mm2/s with a sensitivity of 73% and specificity of 91.7% [26], and Robba et al. [27] who found cut-off ADC value of 1.45 x 10-3mm2/s with a sensitivity of 90.9% and specificity of 60%.

A study conducted by Geneidi et al. with the inclusion of vertebral lesions showed cut-off ADC value of 0.67 x10-3 mm2/sec for differentiating benign and malignant bone tumor with sensitivity of 94% and specificity of 79% [28] while in our study sample without inclusion of vertebral lesions showed cut off mean ADC value of 1.074 x 10-3mm2/s with a sensitivity of 83.3%, specificity of 72.7%. In our study sample, four patients (8.5%) of GCT showed false positivity for malignant bone tumors with ADC value below the cut-off ADC value of 1.058 x 10-3mm2/s. Ashikyan et al. [29] showed GCT had a mean ADC value of 1.0±0.2[SD] x10-3mm2/s while in our study sample it was 1.251±0.342[SD].

Neubauer et al. [30] observed the mean ADC value below 1.03 x 10-3mm2/s was a strong indicator for pediatric musculoskeletal malignancy after using a b-value of 50 and 800 s/mm2. In our study sample, we found mean ADC value below 1.058 x 10-3mm2/s is a good indicator of malignant bone tumor with a sensitivity of 83.3%, specificity of 66.7%, and diagnostic accuracy of 78.7%. So our findings are well correlated with this previous study.

Limitation

Exclusion of various benign bone as well as soft tumors in our study sample and inclusion of most of giant cell tumor of bone limited the mean ADC value results. Vast heterogeneity of the musculoskeletal tumors is a major weakness for DWI imaging and ADC mapping for diagnosis and differentiation of various musculoskeletal tumors. Therefore, a larger study sample size to confirm these findings is warranted in the future.

## Conclusions

Quantitative diffusion-weighted imaging and ADC mapping helps in the evaluation of musculoskeletal tumors in conjunction with conventional MRI sequences. In our study, we found a statistically significant difference of mean ADC value between the benign and malignant bone as well as soft tissue tumors. Even though DWI and ADC mapping alone may not be useful for differentiating various benign and malignant musculoskeletal tumors because of overlapping ADC values. However, mean ADC values may serve as an additional tool while combining with the conventional MRI characteristics to diagnose and differentiate various musculoskeletal tumors.
